# Quantitative DNA Methylation Analysis and Epigenotype-Phenotype Correlations in Taiwanese Patients with Beckwith-Wiedemann Syndrome

**DOI:** 10.3390/jpm11111066

**Published:** 2021-10-22

**Authors:** Hsiang-Yu Lin, Chung-Lin Lee, Sisca Fran, Ru-Yi Tu, Ya-Hui Chang, Dau-Ming Niu, Chia-Ying Chang, Pao Chin Chiu, Yen-Yin Chou, Hui-Pin Hsiao, Chia-Feng Yang, Meng-Che Tsai, Tzu-Hung Chu, Chih-Kuang Chuang, Shuan-Pei Lin

**Affiliations:** 1 Department of Medicine, MacKay Medical College, New Taipei City 25245, Taiwan; lxc46199@ms37.hinet.net (H.-Y.L.); clampcage@yahoo.com.tw (C.-L.L.); 2 Department of Pediatrics, MacKay Memorial Hospital, Taipei 10449, Taiwan; wish1001026@gmail.com; 3 Department of Medical Research, MacKay Memorial Hospital, Taipei 10449, Taiwan; fransiscazen@gmail.com (S.F.); likemaruko@hotmail.com (R.-Y.T.); 4 MacKay Junior College of Medicine, Nursing and Management, Taipei 10449, Taiwan; 5 Department of Medical Research, China Medical University Hospital, China Medical University, Taichung 400, Taiwan; 6 Department of Rare Disease Center, MacKay Memorial Hospital, Taipei 10449, Taiwan; 7 Institute of Clinical Medicine, National Yang-Ming University, Taipei 10449, Taiwan; dmniu1111@yahoo.com.tw; 8 Institute of Clinical Medicine, National Yang-Ming Chiao Tung University, Taipei 10449, Taiwan; 9 Department of Pediatrics, Taipei Veterans General Hospital, Taipei 10449, Taiwan; pum_chia@yahoo.com.tw; 10 Department of Pediatrics, MacKay Memorial Hospital, Hsinchu 300, Taiwan; ailxjdau@yahoo.com.tw; 11 Department of Pediatrics, Kaohsiung Veterans General Hospital, Kaohsiung 800, Taiwan; paochinped333@gmail.com; 12 Department of Pediatrics, National Cheng Kung University Hospital, College of Medicine, National Cheng Kung University, Tainan 70101, Taiwan; yenyin@mail.ncku.edu.tw (Y.-Y.C.); ache93@yahoo.com.tw (M.-C.T.); 13 Department of Pediatrics, Kaohsiung Medical University Chung Ho Memorial Hospital, Kaohsiung 807, Taiwan; hsiaohb@yahoo.com.tw; 14 Children Growth & Development Department, IHMED International Medical, Taipei 108, Taiwan; mno4.chu@gmail.com; 15 College of Medicine, Fu-Jen Catholic University, Taipei 10449, Taiwan; 16 Department of Infant and Child Care, National Taipei University of Nursing and Health Sciences, Taipei 10449, Taiwan

**Keywords:** Beckwith-Wiedemann syndrome, epigenotype, MassARRAY, phenotype, quantitative DNA methylation

## Abstract

Background: Beckwith-Wiedemann syndrome (BWS; OMIM 130650) is a rare overgrowth syndrome with tumor predisposition resulting from the abnormal expression or function of imprinted genes of the chromosome 11p15.5 imprinting gene cluster. The aim of this study was to identify the epigenotype-phenotype correlations of these patients using quantitative DNA methylation analysis. Methods: One hundred and four subjects with clinically suspected BWS were enrolled in this study. All of the subjects had been referred for diagnostic testing which was conducted using methylation profiling of *H19*-associated imprinting center (IC) 1 and *KCNQ1OT1*-associated IC2 in high-resolution melting analysis and methylation quantification with the MassARRAY assay. Correlations between the quantitative DNA methylation status and clinical manifestations of the enrolled subjects were analyzed. Results: Among the 104 subjects, 19 had IC2 hypomethylation, 2 had IC1 hypermethylation, and 10 had paternal uniparental disomy (pUPD). The subjects with IC2 hypomethylation were characterized by significantly more macroglossia but less hemihypertrophy compared to the subjects with pUPD (*p* < 0.05). For 19 subjects with IC2 hypomethylation, the IC2 methylation level was significantly different (*p* < 0.05) between the subjects with and without features including macroglossia (IC2 methylation level: 11.1% vs. 30.0%) and prenatal or postnatal overgrowth (8.5% vs. 16.9%). The IC2 methylation level was negatively correlated with birth weight *z* score (*p* < 0.01, *n* = 19) and birth height *z* score (*p* < 0.05, *n* = 13). For 36 subjects with clinically diagnosed BWS, the IC2 methylation level was negatively correlated with the BWS score (*r* = −0.592, *p* < 0.01). The IC1 methylation level showed the tendency of positive correlation with the BWS score without statistical significance (*r* = 0.137, *p* > 0.05). Conclusions: Lower IC2 methylation and higher IC1 methylation levels were associated with greater disease severity in the subjects with clinically diagnosed BWS. Quantitative DNA methylation analysis using the MassARRAY assay could improve the detection of epigenotype-phenotype correlations, which could further promote better genetic counseling and medical care for these patients.

## 1. Introduction

Beckwith-Wiedemann syndrome (BWS; OMIM 130650) is a congenital epigenetic overgrowth disorder with tumor predisposition caused by an abnormal expression or function of imprinted genes of the chromosome 11p15.5 imprinting gene cluster. It is characterized by a spectrum of clinical features, including macroglossia, macrosomia, omphalocele or umbilical hernia, ear creases or pits, renal abnormalities, facial nevus flammeus, neonatal hypoglycemia, hemihypertrophy, cardiac malformations, polydactyly, cleft palate, intra-abdominal visceral organomegaly, and a 7.5% reported risk of developing embryonal Wilms’ tumor, hepatoblastoma, neuroblastoma, or adrenocortical carcinoma [1,2,3,4,5,6,7,8,9,10]. The incidence of BWS is estimated to be 1:10,000–13,700 live births [11], with an increased risk associated with assisted reproductive technologies (ART) of around 1 in 1100 [12].

The first clinical reports of BWS were described by Beckwith in 1963 and Wiedemann in 1964 [13,14], and subsequent advances have helped define the molecular defects of this disorder with clinical and genetic heterogeneity. BWS is associated with defective genomic imprinting, a process involving a parent-of-origin-specific gene expression. The chromosome 11p15.5 imprinting region harbors two imprinting domains, *IGF2/H19* and *CDKN1C/KCNQ1/KCNQ1OT1*, which are controlled by *H19*-associated imprinting center 1 (IC1) and *KCNQ1OT1*-associated IC2, respectively [2]. *H19*-associated IC1 is methylated on the paternal allele and unmethylated on the maternal allele, whereas *KCNQ1OT1*-associated IC2 is methylated on the maternal allele and unmethylated on the paternal allele. In patients with BWS, hypomethylation at IC2 occurs in 50–60%; paternal uniparental disomy (pUPD) 11p15.5 occurs in 10–20%; hypermethylation at IC1 occurs in 5–10%; and *CDKN1C* mutations occur in 5–10% (in 5% of sporadic cases and in 40% of familial BWS cases) [1,2,4,5,6,7,8,9]. Chromosomal abnormalities including duplications, deletions, and translocations of the 11p15 region have been reported in <5% of patients [15].

According to the diagnostic criteria proposed by Zarate et al. [16], the existence of three major features (macroglossia, prenatal or postnatal overgrowth, and abdominal wall defects) or two major features and one minor feature (e.g., ear creases or pits, facial nevus flammeus, hemihypertrophy, neonatal hypoglycemia, midface hypoplasia, cardiomegaly, renal abnormalities, or polyhydramnios) is required for the clinical diagnosis of BWS. A number of studies have described the clinical and molecular findings of patients with BWS [1,2,3,4,5,8,9,10,17,18,19,20,21,22,23,24]. Phenotype and genotype/epigenotype correlations in European and North American BWS patients have been described in the literature. For instance, omphalocele has been reported to occur more commonly in patients with IC2 hypomethylation or *CDKN1C* point mutations, whereas macroglossia, macrosomia, and an increased risk of embryonic tumors have been more frequently associated with IC1 hypermethylation. Moreover, hemihypertrophy has been significantly associated with uniparental disomy (UPD) [1,20,21,22].

Genetic and epigenetic alterations in BWS can be present in a mosaic condition leading to mild methylation defects. The MassARRAY assay is a sensitive, accurate, and reliable technique for cost-effective high-throughput methylation analysis, and it can help to improve the detection of disease genes and increase our understanding of epigenetic modifications [25]. At present, only a few studies have analyzed quantitative DNA methylation and investigated the epigenotype-phenotype correlations in patients with BWS [17,18]. Therefore, the aim of this study was to identify epigenotype-phenotype relationships in patients with BWS using quantitative DNA methylation analysis with the MassARRAY assay.

## 2. Patients and Methods

### 2.1. Patient Selection

One hundred and four subjects with clinically suspected BWS (60 males and 44 females; ages ranged from 2 days to 28 years) who were referred to our hospital for diagnostic testing from May 2007 through December 2020 were enrolled in this study. All information was acquired from their medical records. A chart review was conducted by a single author (HYL) to ensure consistent extraction of information. Written informed consent was obtained from a parent if the subject was under 18 years and from the patients themselves if they were over 18 years. The study was approved by the Ethics Committee of MacKay Memorial Hospital, Taipei, Taiwan.

### 2.2. Clinical Assessments

Clinical manifestations were recorded according to the diagnostic criteria proposed by Zarate et al. [16], including major features (macroglossia, prenatal or postnatal overgrowth, and abdominal wall defects) and minor features (ear creases or pits, renal abnormalities, facial nevus flammeus, neonatal hypoglycemia, hemihypertrophy, congenital cardiac malformations, neoplasia, polydactyly, cleft palate, and intra-abdominal visceral organomegaly). Ibrahim el al. [5] designed a practical weighted molecular abnormality outcome scoring system to classify patients with the most common features of BWS. We calculated a total diagnostic score for each patient based on the above BWS clinical scoring system (maximum = 8) [5], giving a differentially weighted score in accordance with the existence of the following features: macroglossia (2.5), exomphalos (1.5), organomegaly (1), macrosomia (1), facial nevus flammeus (1), hemihypertrophy (0.5), and hypoglycemia (0.5). Other data obtained from the medical records included gender, history of conception by ART, and birth history (date, gestational age, birth height and weight).

The subjects’ height and weight at diagnosis were also analyzed in addition to their birth height and birth weight. Standard deviation scores (*z* scores) for height and weight were calculated using standard growth tables for the Taiwanese population [26]. A *z* score was derived by subtracting the population mean from each individual’s raw score, and then dividing the difference by the standard deviation of the population.

### 2.3. Molecular Studies

#### DNA Extraction and Bisulfite Treatment

Genomic DNA (gDNA) from the suspected BWS patients was extracted from 5 mL blood obtained from EDTA tubes using a QIAamp DNA Blood Mini Kit (Qiagen, Hilden, Germany) according to the manufacturer’s protocols. The quality and quantity of DNA were checked using a NanoDrop 2000 spectrophotometer (Thermo Fisher Scientific, Wilmington, DE, USA) with the 260/280 ratio within the 1.8–1.9 range. Afterwards, 1 µg of gDNA was treated with bisulfite using a MethylCode™Bisulfite Conversion Kit (Invitrogen, Carlsbad, CA, USA) following the manufacturer’s instructions, and the final product was 20 µL of bisulfite-treated gDNA.

### 2.4. Methylation Analysis Using Methylation-Sensitive High-Resolution Melting

For high-resolution melting analysis, the bisulfite-treated DNA was analyzed using a BIO-RAD CFX Connect™ Real Time System (Bio-Rad Laboratories, Hercules, CA, USA). The process included mixing 10 µL Precision Melt Supermix (Bio-Rad Laboratories, CA, USA), 4 µM forward and reverse primers designed for high-resolution melting, 7.7 µL RNase-free water and 1.5 µL bisulfite-treated DNA. The melting curves were obtained by running at 95 °C for 2 min, 45 cycles of 95 °C for 10 s, 56–59 °C for 40 s and 72 °C for 60 s, and a final heteroduplex formation at 95 °C for 30 s then 60 °C for 1 min, and the plate was read at 72–95 °C in 0.2 °C increments with 10 s/step. All of the steps included positive and negative controls along with the patients’ samples.

### 2.5. Methylation Analysis Using the MassARRAY EpiTYPER Platform

The following step was the amplification of bisulfite-treated DNA with *H19* and *KCNQ1OT1.* The quantitation of DNA methylation was performed using the MassARRAY EpiTYPER platform (Sequenom, San Diego, CA, USA) as previously described [25,27,28]. The amplification of these target genes was done by adding 2.5 µL 10X Advantage^®^ 2 PCR Buffer (Takara Bio USA, Inc., San Jose, CA, USA), 5 µM of forward and reverse primers (Supplementary Table S1), 1 µL Advantage^®^ UltraPure PCR Deoxynucleotide Mix (10 mM each dNTP) (Takara Bio USA, Inc., San Jose, CA, USA), 0.25 µL 50X Advantage^®^ 2 Polymerase Mix (Takara Bio USA, Inc.), 19.25 µL RNase-free water, and 1 µL bisulfite-treated DNA to a total volume of 25 µL. The PCR conditions were 94 °C for 5 min, 35 cycles of 94 °C for 30 s, 58–61 °C for 45 s and 72 °C for 45 s, and a final extension time of 72 °C for 10 min. Positive and negative controls were also included in this process. All PCR products were analyzed by 2.5% agarose gel electrophoresis, stained with SYBER green, and then visualized under a UV trans-illuminator. Each PCR product was then added to 1.7 µL RNase-free water and 0.3 µL shrimp alkaline phosphatase (SAP) and incubated in a thermal cycler for 45 min at 37 °C and 5 min at 85 °C to dephosphorylate deoxynucleotide triphosphates. Transcription and T-cleavage reactions were then conducted by adding reagents provided by Sequenom followed by incubation for 3 h at 37 °C in a thermal cycler. After the addition of a cation exchange resin to remove residual salt from the reactions, 7 nL of the purified MassCLEAVE reaction was loaded onto a matrix pad of a SpectroCHIP (Sequenom). Spectra were acquired using MassARRAY Analyzer 4, and the methylation level was analyzed using MassARRAY EpiTYPER software (version 1.2, San Diego, CA, USA).

All diagnostic examinations were performed by methylation profiling of *H19*-associated IC1 and *KCNQ1OT1*-associated IC2 using high-resolution melting analysis and high-resolution quantitative methylation profiling with a methylation-specific polymerase chain reaction assay using the MassARRAY EpiTYPER platform (Sequenom, San Diego, CA, USA). DNA samples from 100 age-matched healthy controls were included in this study to set up the MassARRAY methylation panel and define the normal range of methylation levels. The concomitant presence of IC1 hypermethylation and IC2 hypomethylation was considered to indicate UPD [17,18].

### 2.6. Data and Statistical Analysis

We compared the clinical features and BWS scores between the subjects with IC2 hypomethylation and pUPD using the Student’s *t*-test for continuous variables and Fisher’s exact test for categorical variables. Correlations between the quantitative DNA methylation status and clinical manifestations of the subjects were analyzed, and two-tailed *p*-values were computed. The relationships between BWS score and IC1 and IC2 methylation levels of the subjects were evaluated using Pearson’s correlation coefficient (*r*), and testing for statistical significance (*p* < 0.05) was carried out using Fisher’s *r-z* transformations. Relationships between IC2 methylation levels and birth weight and birth height *z* scores of the subjects with IC2 hypomethylation were also analyzed. All statistical analyses were conducted using SPSS version 11.5 (SPSS Inc., Chicago, IL, USA), and any differences with a *p* < 0.05 were considered statistically significant.

**Figure 1:**
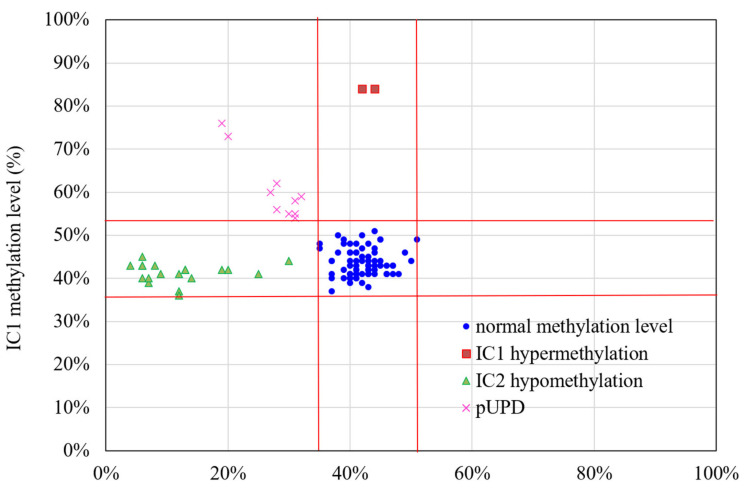
IC1 and IC2 methylation levels in the 104 subjects with suspected Beckwith-Wiedemann syndrome in this study. IC, imprinting center; pUPD, paternal uniparental disomy. * Red lines represent upper and lower limits of the reference ranges (IC1: 36–53%, IC2: 35–51%).

**Figure 2:**
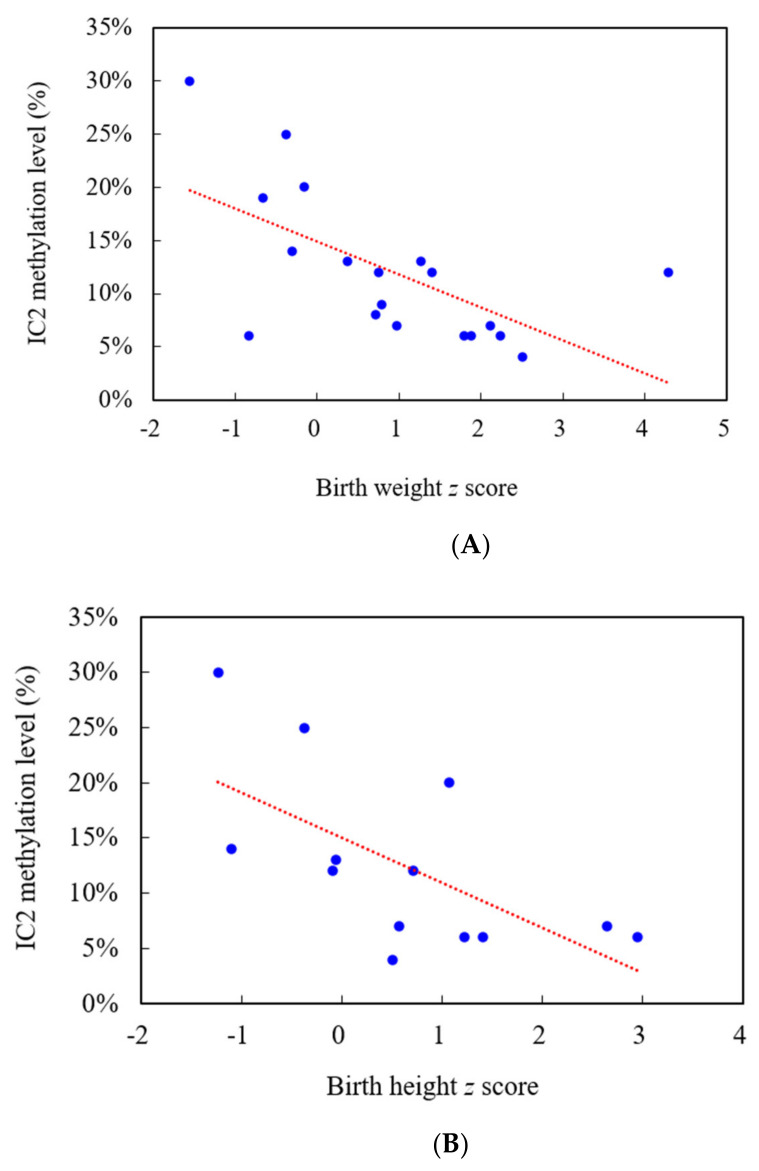
The relationships between IC2 methylation level and *z* scores of birth weight and birth height in the BWS subjects with IC2 hypomethylation. (**A**) Birth weight *z* score (*n* = 19, *r* = −0.617, *p* < 0.01). (**B**) Birth height *z* score (*n* = 13, *r* = −0.639, *p* < 0.05).

**Figure 3:**
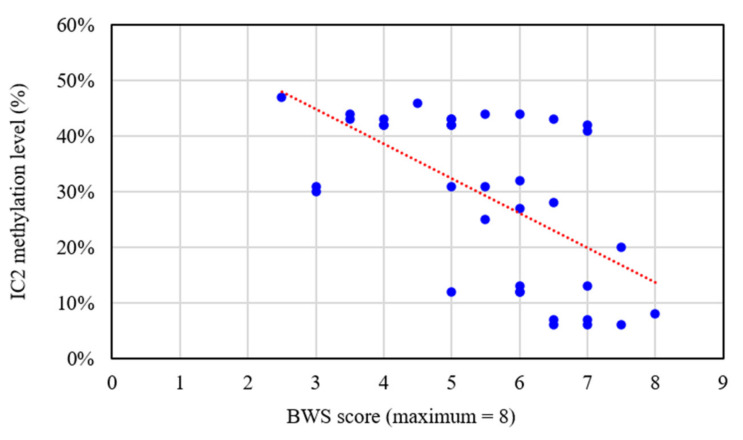
The relationships between IC2 methylation level and BWS score in the 36 subjects with clinically diagnosed BWS in this study (*r* = −0.592, *p* < 0.01).

**Figure 4:**
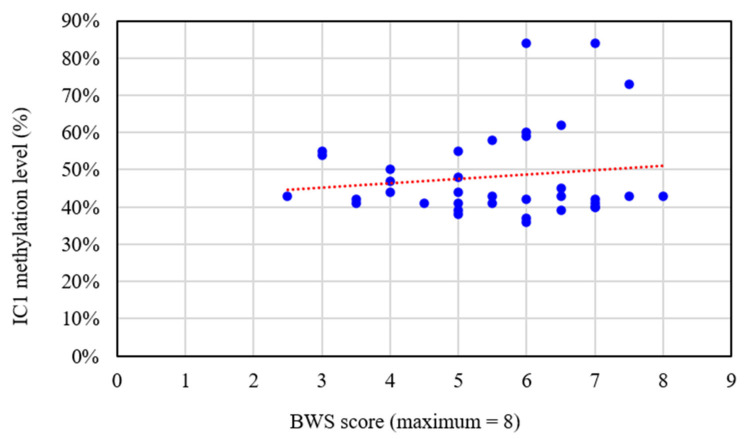
The relationships between IC1 methylation level and BWS score in the 36 subjects with clinically diagnosed BWS in this study (*r* = 0.137, *p* > 0.05).

Among the 104 subjects, 36 were categorized as having a clinical diagnosis of BWS (the presence of three major features or two major features and at least one minor feature), 38 as having suspected BWS (the presence of at least one major feature) [18], and 30 as having only minor feature(s) of BWS. The mean BWS scores (maximum = 8) of these three groups were 5.5, 2.5, and 0.9, respectively. IC2 hypomethylation, IC1 hypermethylation, and pUPD were identified in 19, 2, and 10 of the subjects, respectively. The molecular diagnosis rate was 61% for the subjects with a clinical diagnosis, 18% for those with suspected BWS, and 7% for those with only minor criteria. The molecular defect detection rate was positively correlated with BWS score (*r* = 0.623, *p* < 0.01) (Table 1). Notably, there were two molecularly-positive patients among the 30 subjects with only minor criteria. One patient had Wilms’ tumor identified with IC2 hypomethylation. Another patient had left limb hemihypertrophy and left nephromegaly identified with pUPD. Figure 1 shows the IC1 and IC2 methylation levels for the 104 subjects. The subjects with IC2 hypomethylation (*n* = 19) were characterized by significantly more macroglossia (95% vs. 60%, *p* = 0.018), but less hemihypertrophy (21% vs. 90%, *p* < 0.0001) compared to the subjects with pUPD (*n* = 10). The 19 subjects with a diagnosis of IC2 hypomethylation had a mean BWS score of 5.3, compared to 4.7 in the 10 subjects with pUPD and 6.5 in the two subjects with IC1 hypermethylation. Among the 104 subjects, 11 (11%) were conceived by ART. Of these subjects, three had IC2 hypomethylation (mean BWS score = 6.3), one had pUPD (BWS score = 5.5), and the other seven had normal molecular study results with a mean BWS score of 1.3 (Table 2). In this cohort of 104 subjects, there were 2 individuals with neoplasia. One patient had Wilms’ tumor identified with IC2 hypomethylation. Another patient had pancreatic neck tumor identified with negative molecular result. For 19 subjects with IC2 hypomethylation, the IC2 methylation level was significantly different (*p* < 0.05) between the subjects with and without features including macroglossia (IC2 methylation level: 11.1% vs. 30.0%), prenatal or postnatal overgrowth (8.5% vs. 16.9%), and neoplasia (30.0% vs. 11.1%) (Table 3). Table 4 shows the clinical characteristics and methylation levels of IC1 and IC2 in the 19 subjects with IC2 hypomethylation. In these 19 subjects, the IC2 methylation level was also negatively correlated with their birth weight *z* score (*p* < 0.01, *n* = 19) and birth height *z* score (*p* < 0.05, *n* = 13) (Figure 2A,B). For 36 subjects with clinically diagnosed BWS, the IC2 methylation level was negatively correlated with the BWS score (*r* = −0.592, *p* < 0.01) (Figure 3). The IC1 methylation level showed the tendency of positive correlation with the BWS score without statistical significance (*r* = 0.137, *p* > 0.05) (Figure 4). The IC1 methylation level was higher for the subjects with features of pre- or postnatal overgrowth (IC1 methylation level: 48.9% vs. 41.0%) and hemihypertrophy (52.2% vs. 46.0%) than those without these features with no statistical significance (*p* > 0.05) (Table 5).

## 3. Discussion

To the best of our knowledge, this is the first cohort study to analyze quantitative DNA methylation using the MassARRAY assay and investigate the epigenotype-phenotype correlations in clinically diagnosed BWS subjects in Taiwan. We used the MassARRAY assay to analyze methylation levels at the IC1 and IC2 loci of 11p15.5, and found that lower IC2 methylation and higher IC1 methylation levels were associated with greater disease severity in clinically diagnosed BWS subjects. The subjects with IC2 hypomethylation were characterized by significantly more macroglossia but less hemihypertrophy compared to those with pUPD. Our results are consistent with those of previous studies [5,18]. Of those cases with clinical diagnosis of BWS (*n* = 36), there were 14 cases (39%) with unknown epigenetic or genetic defects supporting that a group of molecular assays are necessary to define the genotype-phenotype correlations.

Using the MassARRAY assay, we confirmed the diagnosis of BWS in 61% of 36 subjects with a clinical diagnosis, 18% of 38 subjects with suspected BWS, and 7% of 30 subjects with only minor criteria. The molecular diagnosis rates in this study are consistent with those reported by Calvello et al. [18]. This indicates that the MassARRAY assay is a reliable test to confirm clinically suspected BWS. In this study, for 38 subjects with only one major feature of BWS, the molecular diagnosis rate was still as high as 18%, and thus molecular studies are needed to confirm the molecular defects for these patients.

Among the 31 BWS patients with molecular defects in our cohort, the frequencies of the different methylation defects of IC2 hypomethylation, IC1 hypermethylation, and pUPD were 61%, 6% and 32%, respectively, which are in agreement with those reported in the literature (66%, 7%, and 27% respectively) [18].

In this study, we used methylation-sensitive high-resolution melting analysis, which has been reported to be a rapid, cost-effective and sensitive method for screening mosaic methylation changes at the *KCNQ1OT1* and *H19* loci in BWS [29,30]. In addition, we used the MassARRAY EpiTYPER mass spectrometer analysis technology platform. Accurate analysis of methylation at the imprinting control regions of 11p15.5 is an important tool for the molecular diagnosis of BWS. The MassARRAY assay can more accurately analyze methylation variations of nucleic acids compared with the lower accuracy of qualitative (methylation-specific PCR) and semi-quantitative (southern blotting and methylation-sensitive multiplex ligation probe analysis) methods [27]. The high sensitivity of this method is particularly suitable to identify UPD, which can be underestimated when using other qualitative and semi-quantitative analyses. Calvello et al. [18] reported that genetic and epigenetic alterations may be present in a mosaic pattern and can thereby lead to mild methylation defects. Since the UPD type of patients with BWS have a higher risk of cancer, neglecting UPD may be an important limitation for the recognition of BWS [18,31]. In addition, this method can be used to identify UPD without the need to perform microsatellite analysis of the parents’ DNA, which can help to increase the diagnostic rate.

Calvello et al. [18] reported that in their patients with IC1 hypermethylation (with normal IC2 methylation), there was a correlation (*p* < 0.001) between the percentage of methylation and clinical BWS features including macroglossia, macrosomia, visceromegaly, and abdominal wall defects. They suggested that there was a direct association between the percentage of methylation and the severity of BWS, which is consistent with our findings.

Lee et al. [17] reported that IC2 methylation scores quantified by methylation-specific pyrosequencing were negatively correlated with the birth weight and birth height of their patients with BWS (*n* = 18) and Silver-Russell syndrome (*n* = 20). Similarly, in our 19 subjects with IC2 hypomethylation, the IC2 methylation level was also negatively correlated with their birth weight *z* score and birth height *z* score.

The imprinted genes on 11p15.5 are thought to be critical for renal development. The prevalence of nephro-urological anomalies in BWS has been reported to range from 28–61% [32]. Goldman et al. [32] described that of 159 patients with BWS, 67 (42%) exhibited renal abnormalities, including nephromegaly (25%), collecting system abnormalities (11%), and renal cysts (10.5%). Similarly, among our 36 clinically diagnosed BWS patients, 17 (47%) exhibited renal abnormalities, of which nephromegaly (25%) was the most common finding, followed by renal cysts (8%). Mussa el al. [33] reported that 56% of their BWS patients had nephro-urological abnormalities which were mostly associated with the IC1 and UPD subtypes, and that nephromegaly/hyperplasia was the most common and severe finding (36.5%) in the IC1 patients. In our cohort, renal anomalies were also more frequently observed in the IC1 (100%) and UPD (50%) subtypes than in IC2 (26%) subtype. In addition, the IC1 methylation level was higher in the subjects with renal anomalies than in those without renal anomalies (49.6% vs 47.0%). Our results are consistent with previous studies.

Congenital heart disease is more prevalent in patients with BWS than in the general pediatric population, and cardiac defects have been reported in up to 13–20% of patients with BWS [8,24]. Similarly, among our 36 clinically diagnosed BWS patients, 10 (28%) had cardiac defects and interatrial or interventricular defects (22%) were the most common findings. Minor anatomical defects should be monitored by echocardiography until spontaneous resolution, but more severe defects may need surgical correction similar to that in sporadic cases of congenital heart disease [9].

Mussa et al. [22] reported different prevalence rates of the clinical features in patients with various molecular subtypes of BWS. They found that hemihypertrophy was more common in those with UPD, but that the three major features of macroglossia, macrosomia, and abdominal wall defects were less common in those with UPD compared to the other molecular subtypes. Consistent with their findings, UPD was less likely to lead to the typical major features of BWS. In our cohort, hemihypertrophy was also more common and macroglossia was less common in the subjects with UPD than in those with the IC2 hypomethylation or IC1 hypermethylation subtypes. In the BWS scoring system developed by Ibrahim el al. [5], the three major features have a total of 5 points, compared to only 0.5 points for hemihypertrophy. This may explain why the mean BWS score of the patients with pUPD (4.7) was lower than those of the patients with IC2 hypomethylation (5.3) and IC1 hypermethylation (6.5).

ART, such as in vitro fertilization (IVF) and intracytoplasmic sperm injection (ICSI), may impact the establishment and/or the maintenance of DNA methylation at imprinted loci, and it has been associated with epigenetic disorders such as BWS, Silver–Russell syndrome, Prader–Willi syndrome, and Angelman syndrome [34,35,36,37]. Mussa et al. [12] reported a 10-fold increased risk of BWS with ART and an absolute risk of about 1 in 1100. More than 90% of children with molecularly confirmed BWS conceived by ART have IC2 hypomethylation. Previous studies have reported that about 4.0–13.4% of patients with BWS are conceived by ART [12,37,38,39]. In our cohort, four clinically diagnosed patients with BWS (4/36, 11%) were conceived by ART. IC2 hypomethylation and pUPD were detected in three and one of these patients, respectively. Our results are in agreement with the previous reports. The complicated molecular findings underlying BWS are challenging for both geneticists counseling affected families and laboratories offering these tests. To provide genetic counseling to families with BWS, the knowledge of the nature of the epimutation or mutation subtype is important to delineate exact risk figures, and genetic counseling by an experienced clinical geneticist is required [40].

### Limitations

Due to the limited sample size in this single-center study, we were not able to draw strong conclusions about the individual major and minor features among BWS patients with different molecular defects or their phenotypic effects. Data on *CDKN1C* point mutations and microdeletion/microduplication of chromosome 11p15.5 were not available due to the limitations of the study design. However, the small sample size of patients with different molecular defects of BWS also reflects the rare nature of this genetic disease. Consequently, further studies with larger cohorts and longer follow-up periods are warranted to validate our findings. 

## 4. Conclusions

Lower IC2 methylation and higher IC1 methylation levels were associated with greater disease severity of the subjects with clinically diagnosed BWS. The subjects with IC2 hypomethylation were characterized by significantly more macroglossia but less hemihypertrophy compared to the subjects with pUPD. In the subjects with IC2 hypomethylation, there was a significant correlation between the methylation status of IC2 with their birth anthropometric profiles. Quantitative DNA methylation analysis using the MassARRAY assay can improve the detection of epigenotype-phenotype correlations, which can further promote better genetic counseling and medical care for these patients.

## Figures and Tables

**Table 1:** Epigenetic defects of the 36 subjects with clinically diagnosed BWS, 38 subjects with suspected BWS, and 30 subjects with only minor features of BWS.

Clinical Classification	BWS Score (Maximum = 8)	Epigenetic Defects				
		IC2 Hypomethylation (%)	IC1 Hypermethylation (%)	pUPD (%)	Unknown (%)	Molecular Diagnosis Rate
Clinical diagnosis (*n* = 36)	5.5 ±1.4	12 (33%)	2 (5%)	8 (22%)	14 (39%)	61%
Suspected BWS (*n* = 38)	2.5 ± 1.0	6 (16%)	0	1 (3%)	31 (82%)	18%
All (*n* = 74)	4.0 ± 1.9	18 (24%)	2 (3%)	9 (12%)	45 (61%)	39%
Only minor criteria (*n* = 30)	0.9 ± 0.5	1 (3%)	0	1 (3%)	28 (93%)	7%

BWS, Beckwith-Wiedemann syndrome; IC, imprinting center; pUPD, paternal uniparental disomy.

**Table 2:** Clinical features of the 19 subjects with IC2 hypomethylation, 10 subjects with pUPD, and 2 subjects with IC1 hypermethylation.

Clinical Features	IC2 Hypomethylation (*n* = 19)	pUPD (*n* = 10)	*p* Value	IC1 Hypermethylation (*n* = 2)
BWS score (maximum = 8)	5.3 ± 1.9	4.7 ± 2.0	0.646	6.5 ± 0.7
Assisted reproductive technology	3 (16%)	1 (10%)	0.681	0
Major features				
Macroglossia	18 (95%)	6 (60%)	0.018	2 (100%)
Pre- or postnatal gigantism (growth >90th centile)	11 (58%)	9 (90%)	0.081	2 (100%)
Abdominal wall defects	11 (58%)	6 (60%)	0.917	2 (100%)
Minor features				
Ear creases/pits	11 (58%)	3 (30%)	0.164	2 (100%)
Renal abnormalities	5 (26%)	5 (50%)	0.216	2 (100%)
Facial naevus flammeus	10 (53%)	3 (30%)	0.260	1 (50%)
Neonatal hypoglycemia	5 (26%)	0	0.079	0
Hemihypertrophy	4 (21%)	9 (90%)	0.0001	0
Congenital cardiac malformations	5 (26%)	1 (10%)	0.32	1 (50%)
Neoplasia	1 (5%)	0	0.478	0
Moderate or severe mental retardation	2 (11%)	0	0.305	0
Polydactyly	0	0	—	0
Cleft palate	0	0	—	0
Intra-abdominal visceral organomegaly	13 (68%)	6 (60%)	0.664	2 (100%)

BWS, Beckwith-Wiedemann syndrome; IC, imprinting center; pUPD, paternal uniparental disomy. *p* < 0.05 are printed in bold.

**Table 3:** Quantitative IC2 methylation level by MassARRAY for 19 BWS subjects with IC2 hypomethylation in this study with or without each major and minor BWS features.

Major and Minor Features	With or Without Certain Features	N	*Mean IC2 Methylation Level (%)	*p* Value
Major features				
Macroglossia	With	18	11.1	**0.005**
	Without	1	30.0	
Pre- or postnatal overgrowth (growth >90th centile)	With	11	8.5	**0.007**
	Without	8	16.9	
Abdominal wall defect	With	11	10.5	0.258
	Without	8	14.3	
Minor features				
Ear creases/pits	With	11	9.9	0.123
	Without	8	15.0	
Renal abnormalities	With	5	11.2	0.763
	Without	14	12.4	
Facial naevus flammeus	With	10	12.0	0.974
	Without	9	12.1	
Neonatal hypoglycemia	With	5	10.4	0.557
	Without	14	12.6	
Hemihypertrophy	With	4	12.0	0.987
	Without	15	12.1	
Congenital cardiac malformations	With	5	12.4	0.709
	Without	14	11.0	
Neoplasia	With	1	30.0	**0.005**
	Without	18	11.1	
Moderate/severe mental retardation	With	2	7.0	0.297
	Without	17	12.6	
Polydactyly	With	0	—	1.000
	Without	19	12.1	
Cleft palate	With	0	—	1.000
	Without	19	12.1	
Intra-abdominal visceral organomegaly	With	13	11.2	0.470
	Without	6	13.8	

IC, imprinting center; BWS, Beckwith-Wiedemann syndrome. *p* < 0.05 are printed in bold. * Reference range: 35–51%.

**Table 4:** Birth characteristics and methylation levels of IC1 and IC2 in the 19 BWS subjects with IC2 hypomethylation.

No.	Gender	Gestational Age (Weeks)	Birth Weight *z* Score	Birth Height *z* Score	BWS Score (Maximum = 8)	IC1 Methylation Level *	IC2 Methylation Level **
1	F	32	0.71	NA	8	43%	8%
2	M	33	1.79	1.22	7.5	43%	6%
3	M	38	2.23	2.94	7	40%	6%
4	M	32	0.97	0.57	6.5	39%	7%
5	F	40	1.40	NA	6	36%	12%
6	M	31	−0.38	−0.37	5.5	41%	25%
7	F	39	0.75	0.71	5	41%	12%
8	F	36	−0.31	−1.11	4.5	40%	14%
9	F	37	−0.67	NA	3	42%	19%
10	M	35	0.79	NA	4	41%	9%
11	F	38	−1.56	−1.24	1	44%	30%
12	M	38	−0.83	NA	2.5	43%	6%
13	M	39	2.11	2.65	7	40%	7%
14	F	41	−0.16	1.07	3.5	42%	20%
15	F	38	1.27	−0.06	7	42%	13%
16	M	35	4.28	−0.10	6	37%	12%
17	M	36	1.88	1.40	6.5	45%	6%
18	F	40	2.51	0.50	3.5	43%	4%
19	F	40	0.37	NA	6	42%	13%

Reference ranges: * 36–53%; ** 35–51%. BWS, Beckwith-Wiedemann syndrome; IC, imprinting center; NA, not available.

**Table 5:** Quantitative IC1 methylation level by MassARRAY for 36 subjects with clinical diagnosis of BWS in this study with or without each major and minor BWS features.

Major and Minor Features	With or Without Certain Features	N	* Mean IC1 Methylation Level (%)	*p* Value
Major features				
Macroglossia	With	31	48.2	0.945
	Without	5	48.6	
Pre- or postnatal overgrowth (growth >90th centile)	With	33	48.9	0.283
	Without	3	41.0	
Abdominal wall defects	With	29	48.7	0.638
	Without	7	46.3	
Minor features				
Ear creases/pits	With	20	47.9	0.849
	Without	16	48.7	
Renal abnormalities	With	17	49.6	0.519
	Without	19	47.0	
Facial naevus flammeus	With	14	49.4	0.795
	Without	19	48.3	
Neonatal hypoglycemia	With			
	Without			
Hemihypertrophy	With	13	52.2	0.147
	Without	23	46.0	
Congenital cardiac malformations	With	12	46.2	0.472
	Without	24	49.3	
Neoplasia	With	0	—	1.000
	Without	36	48.3	
Moderate/severe mental retardation	With	4	49.0	0.319
	Without	32	42.5	
Polydactyly	With	1	41.0	0.550
	Without	35	48.5	
Cleft palate	With	1	41.0	0.550
	Without	35	48.5	
Intra-abdominal visceral organomegaly	With	20	49.6	0.478
	Without	16	46.6	

IC, imprinting center; BWS, Beckwith-Wiedemann syndrome. * Reference range: 36–53%.

## Data Availability

Not applicable. There are no other supporting data and materials since all of them are in this article.

## Supplementary Materials

The following are available online at https://www.mdpi.com/article/10.3390/jpm11111066/s1. Table S1. Primer sequences for bisulfite-PCR.

## List of Abbreviations

BWS	Beckwith–Wiedemann syndrome
ART	assisted reproductive technology
IC	imprinting center
pUPD	paternal uniparental disomy
UPD	uniparental disomy
