# Supplementary material for: Quantitative DNA Methylation Analysis and Epigenotype-Phenotype Correlations in Taiwanese Patients with Beckwith-Wiedemann Syndrome
Source: J Pers Med. 2021 Oct 22;11(11):1066. doi: 10.3390/jpm11111066 (PMC8622080; doi:10.3390/jpm11111066)
Supplement: Supplementary file 1 [file jpm-11-01066-s001.zip › jpm-1411009-supplementary.pdf]

SUPPLEMENTARY TABLE

Table S1. Primer sequences for bisulfite-PCR.

Sequence Description	Tm	Sequence	Size	Number of CpG investigated
H19Amp10-10F	61℃	aggaagagagGAGATTTGAGGTGAATTTTAGGGA	337bp	20
H19Amp10-T7R		cagtaatacgactcactatagggagaaggctCAAAACAAAATCCCCACAACC		
KCNQ10T1-Amp1-10F	61℃	aggaagagagTGGTAGGATTTTGTTGAGGAGTTTT	344bp	27
KCNQ10T1-Amp1-T7R		cagtaatacgactcactatagggagaaggctCTCACACCCAACCAATACCTCATAC		
